# Bilateral Cochlear Implant Processing of Coding Strategies With CCi-MOBILE, an Open-Source Research Platform

**DOI:** 10.1109/taslp.2023.3267608

**Published:** 2023-04-17

**Authors:** Ria Ghosh, John H. L. Hansen

**Affiliations:** Center for Robust Speech Systems, CILab – Cochlear Implant Processing Lab, Department of Electrical and Computer Engineering, University of Texas at Dallas, Richardson, TX 75080 USA; Center for Robust Speech Systems, CILab – Cochlear Implant Processing Lab, Department of Electrical and Computer Engineering, Erik Jonsson School of Engineering and Computer Science, University of Texas at Dallas, Richardson, TX 75080 USA, and also with the School of Behavioral and Brain Sciences, University of Texas at Dallas, Richardson, TX 75080 USA

**Keywords:** Cochlear implant (CI), coding strategies, temporal offset, Interaural time duration (ITD), bilateral CIs, ILD, channel synchronization

## Abstract

While speech understanding for cochlear implant (CI) users in quiet is relatively effective, listeners experience difficulty in identification of speaker and sound location. To assist for better residual hearing abilities and speech intelligibility support, bilateral and bimodal forms of assisted hearing is becoming popular among CI users. Effective bilateral processing calls for testing precise algorithm synchronization and fitting between both left and right ear channels in order to capture interaural time and level difference cues (ITD and ILDs). This work demonstrates bilateral implant algorithm processing using a custom-made CI research platform - CCi-MOBILE, which is capable of capturing precise source localization information and supports researchers in testing bilateral CI processing in real-time naturalistic environments. Simulation-based, objective, and subjective testing has been performed to validate the accuracy of the platform. The subjective test results produced an RMS error of ±8.66° for source localization, which is comparable to the performance of commercial CI processors.

## I. Introduction

The cochlear^1^ implant (CI) is a surgical device that is capable of restoring hearing functionality for individuals with severe to profound hearing loss. A receiver coil and a set of 16 to 24 electrodes (depending upon the CI manufacturer) comprise the implanted part of the CI. These electrodes mimic the functionality of the human hair follicles, that involves converting incoming analog sound signals into electric signals for auditory nerve processing. An external behind-the-ear (BTE) processor captures incoming audio and converts it into digital biphasic pulse trains through a signal processing routine [1], [2], [3], [4]. This routine (see [5] for more detail) is based on a relationship between the amplitude/frequency components of speech and the intracochlear electrodes. Information about the number of electrodes to be excited and the desired current levels are encoded in the biphasic pulse train, which then stimulates the implanted electrode array connected to the auditory nerve endings. Specifically, the BTE processor allows the signal to be passed through a set of bandpass filters with frequency allocations equivalent to the tonotopic nature of the cochlea and then rectified to analyze the envelope spectrum. Logarithmic compression is then applied to map the power spectral densities to clinical (auditory based) units for each electrode. Stimulation of electrodes occurs in a continuous, interleaved manner based on biphasic pulse generation from the timing and amplitude range set from the cochlear implant recipient’s electric sensitivity thresholds (also known as a MAP) [6].

The main difference between the biological process of auditory nerve stimulation and the implant triggered artificial stimulation is that there are millions of hair follicles in a healthy human ear controlling the wide frequency of audio signals whereas a cochlear implant only has a max of 24–30 electrodes that can be implanted in the ear for capturing and translating the entire audio frequency. For that reason, even after successful cochlear implantation in one ear, certain CI users have issues in identifying the direction of sound and communicating properly as they tend to continue losing their substantial residual hearing in their better non-implanted ear. Also, transitioning to bilateral CIs when understanding speech with hearing aids (HA) becomes extremely residual, has been found to be highly beneficial to both bilateral and bimodal HA users [7], [8].

Previous studies have reported impression of improved localization ability as well as better perception of speech by bilateral cochlear implantees (BiCIs) [9], [10]. Certain cases like, minor differences in the surgical process during simultaneous implantation, time intervals between the sequential cochlear implantation, possibility of the residual hearing loss due to failure of surgery, mismatch in manufacturer types for each implant, are some factors that might lead to performance deterioration of CI users. To make the adjustment process better, a few approaches have been suggested for better optimization of the fittings in such cases [11], [12], [13], [14], but none of them have been accepted as a best practice yet. After the surgery, factors like adding required delay in the electrical stimulations for synchronous neural activation, optimizing frequency-to-electrode allocation and obtaining equal loudness amplification for both ears play an important role in providing the CI/HA user with a smooth user experience. Various experiments, to determine speech recognition and localization abilities of BiCIs in comparison to normal hearing (NH) subjects, have been conducted by research labs to help audiologists in making the best fittings possible for each user, but the performance of the subjects have been widely varying with no set pattern of improvement [15], [16], [17], [18]. It has been found that, bimodal hearing users with sufficient residual hearing in the ear with a HA, are more capable in detecting direction of sound as they have a slightly wider range of frequency access from their natural capability to hear, as compared to BiCIs with complete hearing loss [8], [9], [19]. A major obstruction to accurate source localization by bimodal and bilateral CI users is the distortion of interaural time and level difference cues (ITD and ILD), and limited ITD sensitivity [20]. A number of CI processing strategies involving encoding of the time differences [21], [22] have been developed to resolve this issue to make the experience of hearing as natural as possible to BiCIs. But most of these algorithm advancements present simulated results and have not been verified with subjective results as it is not possible to alter or add the researcher designed-novel processing, on a CI user’s clinical processor while subject testing. This ends up restricting most of the algorithm improvement and testing to only the CI manufacturers who have access to such resources.

**Fig. 1.**
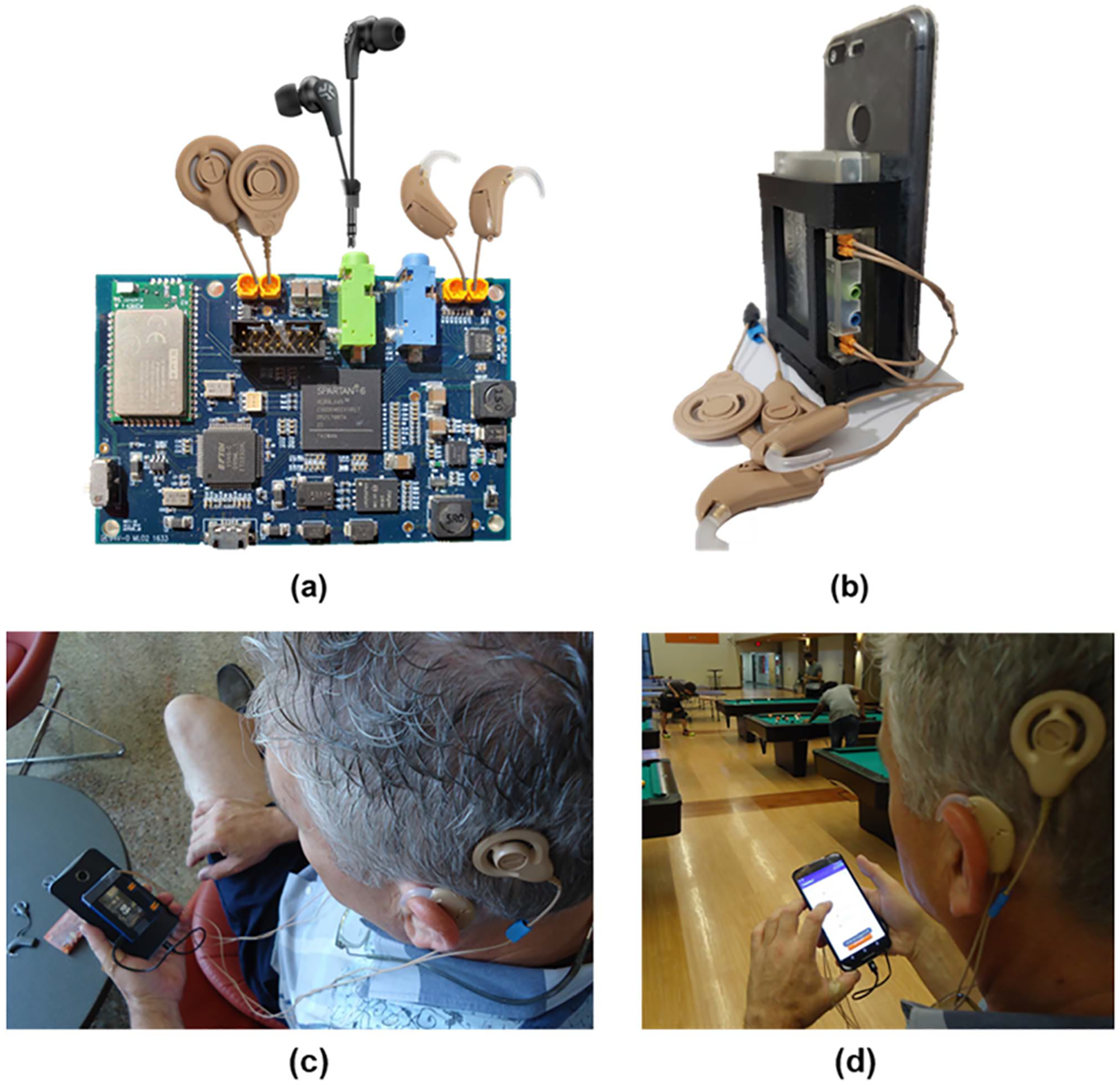
(a). CCi-MOBILE research platform (b) compact portable casing for CCi-MOBILE to fit behind a smart phone, supporting in-field testing in naturalistic environments (c) & (d) subject testing of novel bimodal/bilateral algorithms in naturalistic environments using the CCi-MOBILE.

The growing need of advanced sound coding strategies for bilateral CI users, calls for the requirement of a unified sound processing system, that could give engineers and stand-alone research labs the flexibility to test the progress of their novel algorithms at regular intervals through subjective analysis in naturalistic environments, and not just have simulated probabilistic results. This motivation led to the development of CCi-MOBILE, a custom-made, open-source research platform developed at the Cochlear Implant Laboratory, University of Texas at Dallas. This work presents objective and subject validation along with flexible customization of bilateral and bimodal signal processing algorithms using CCi-MOBILE (Fig. 1(a)). Previous research platforms developed and discussed in [23] required additional resources or externally coordinated hardware synchronization to test algorithms, making them difficult to be used in naturalistic environments for real-time testing. CCi-MOBILE aimed at overcoming such drawbacks and has been designed to be portable and wearable, allowing performance analysis of bilateral strategies in real-time with in-field environments mimicking daily life scenarios for CI/HA users (Fig. 1(c) & (d)). A new bilateral coding strategy discussed in [24] uses the CCi-MOBILE to test accuracy and validity of the strategy with subjects.

## II. CCi-MOBILE Platform

To aid the requirement of optimizing algorithms that run on the CI signal processing (DSP) chip, CCi-MOBILE mimics the external system of a cochlear implant, by replacing the industrybuilt DSP speech processor with a custom-made Printed Circuit Board (PCB) programmed by a reconfigurable Spartan-6 FPGA that can run any novel algorithm developed by researchers. Behind-the-ear (BTE) microphones and RF (radio frequency) coils of the clinical processor are replaced with custom-made microphone shells and RF coils compatible with the PCB, used during subject testing (Fig. 1(b)). The main processing of implementing and modifying the algorithm is performed backend, on the PC/smartphone whereas the FPGA performs the function of real-time/off-line streaming of the input data, analog audio to digital signal conversion and converting back the processed electric pulse into amplified audio (for HAs) or a train of biphasic pulses (for CIs), which is the only form of accepted input to the implanted electrode array. Researchers can perform subject tests using the CCi-MOBILE system to optimize the performance of algorithms programmed in MATLAB and JAVA, prior to going through FDA approvals and executing the very costly FPGA to DSP IC conversion needed to fit the algorithm in a BTE processor. More details are discussed in [25].

## III. Bilateral Processing With CCi-MOBILE

As bilateral cochlear implantation has contributed towards restoring binaural hearing [26], [27], [28], allowing the CI user’s hearing experience to be more natural, it is important to ensure proper synchronization between the left and right output channels to maintain localization of the incoming sound. Audio received by the BTEs connected to the CCi-MOBILE is sampled and digitized by the on-board ADC at the rate of 16 KHz. To support real-time processing and ensure that both left and right channel data transfer is synchronized, the audio samples are buffered for 8 ms (128 samples) before it is passed on to the computing platform (PC/smartphone) for processing in MATLAB or JAVA. The left and right input channels process the incoming audio independently, but the output of both channels is synchronized against the same clock.

**Fig. 2.**
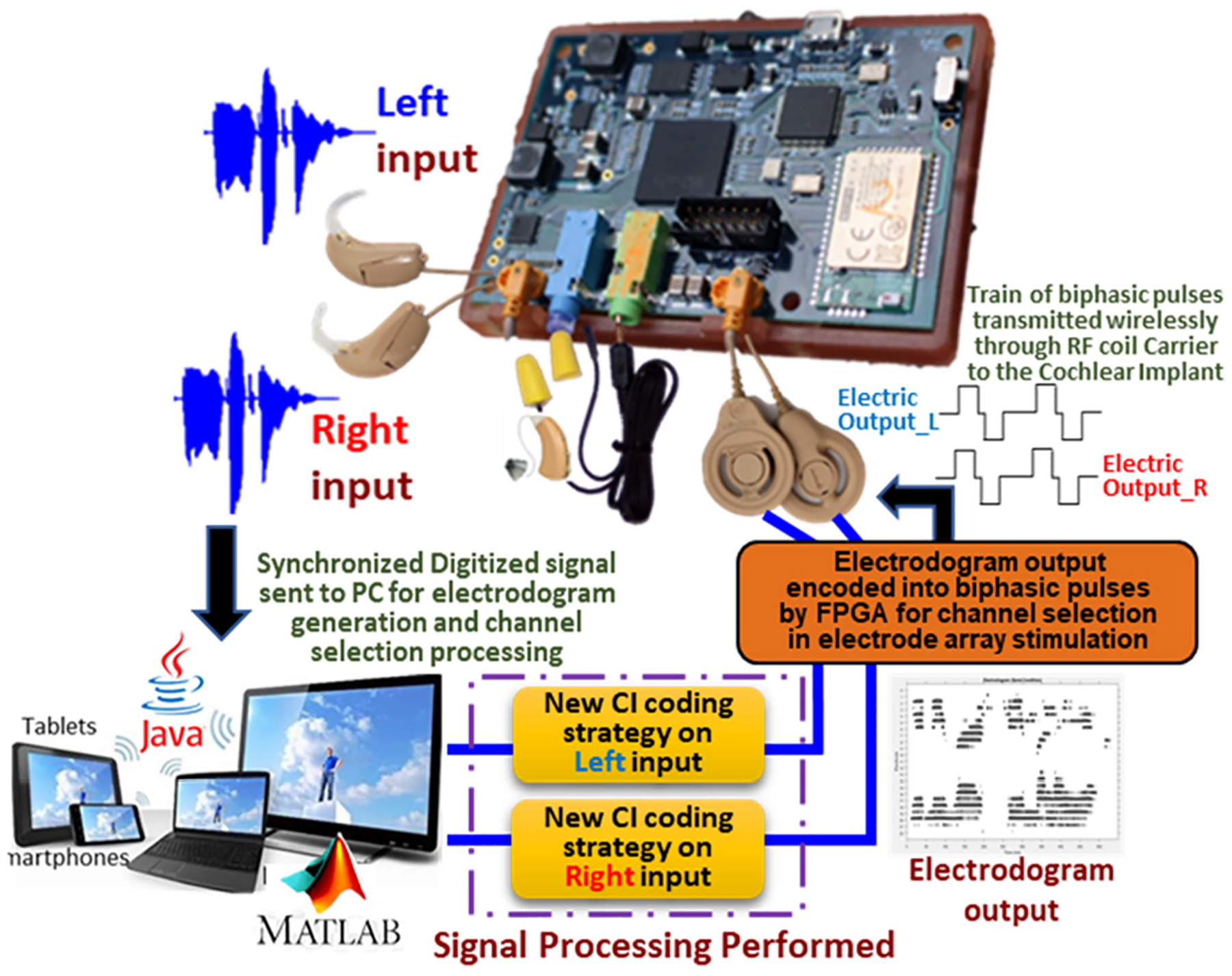
Back-end signal processing performed on each channel independently with electrodogram generation.

The coding strategy programmed by the researcher in MATLAB/JAVA would generate an electrodogram which provides a simulated visual of which electrodes in the electrode array would be the best to be excited at a given point in time (see Fig. 2) This important information of which, when and at what current level would an electrode be excited is decided by the coding strategy developed, allowing the researcher to analyze the effectiveness of their algorithm. The information gets transferred in form of a bit stream to the FPGA which then transmits it wirelessly encoded in a train of biphasic pulses, at user defined stimulation rates through the RF coils to the RF receiver implanted underneath the ear at a RF carrier frequency of 5 MHz.

### A. Sound Coding Strategies With CCi-MOBILE

This work discusses the localization performance with 3 CI sound coding strategies – ACE (Advanced Combinational Encoder), CIS (continuous interleaved sampling), and FACE (formant-based-ACE), using CCi-MOBILE. Each strategy is programmed in a certain way to fit the input and output requirements of CCi-MOBILE. All three coding strategies are compatible with Cochlear Corporation’s Nucleus series of CIs. After implementation, each strategy is analyzed individually and the performance of two different strategies, implemented simultaneously on the left and right channel with varied environmental conditions, is evaluated to make sure that the output still remains synchronized.

#### 1) Continuous-Interleaved Sampling (CIS) Coding Strategy:

**Fig. 3.**
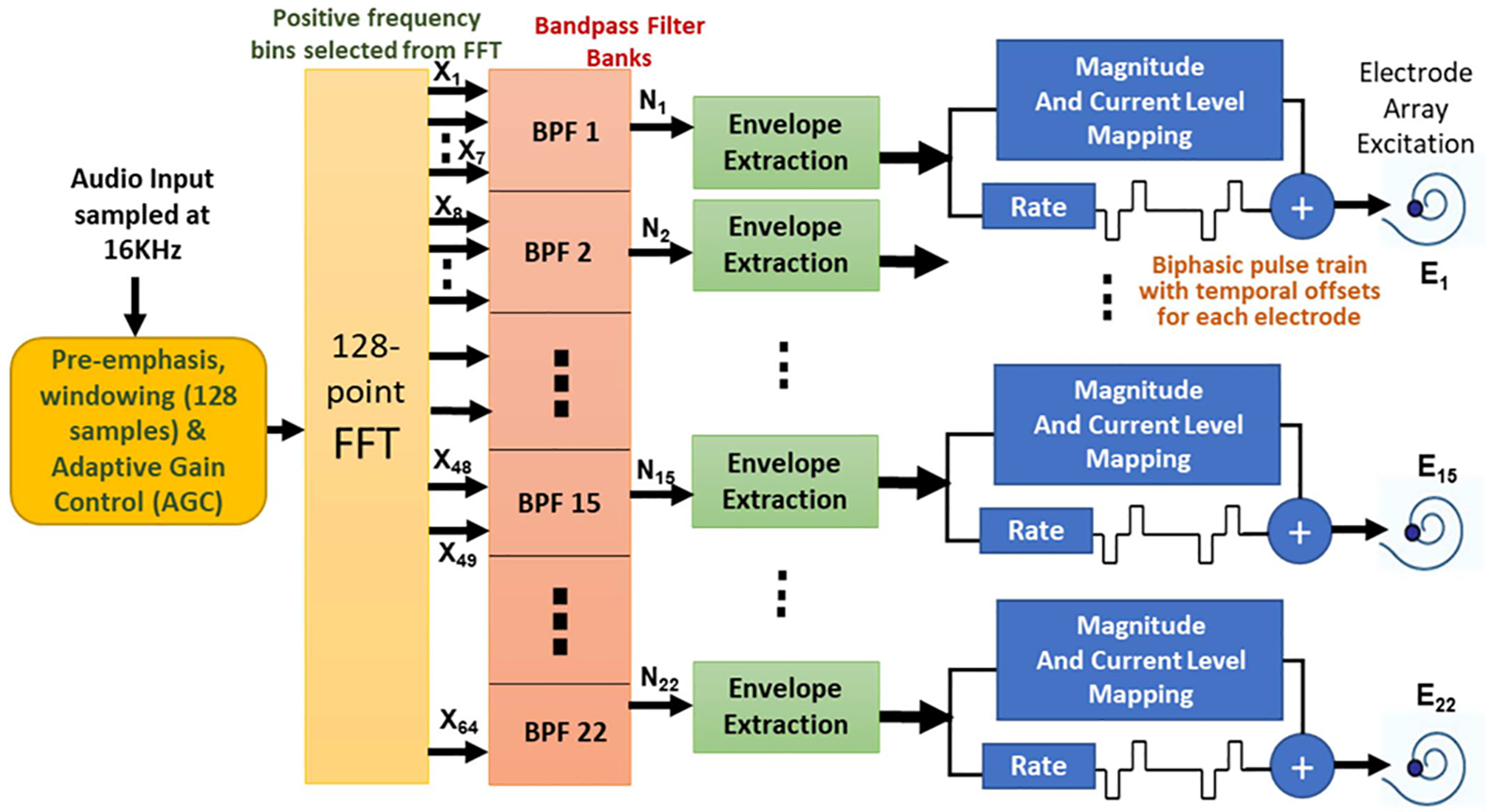
CIS coding strategy block flow used with CCi-MOBILE.

The CIS strategy proposed in [6], [29] ensures efficient channel interaction through interleaved non-simultaneous stimuli. The biphasic pulse train delivered to each electrode has temporal offsets which eliminate any overlap across channels preventing possibilities of over-stimulation of electrodes or current leakage. CIS allows a maximum stimulation rate of 14400 Hz, distributed among all electrodes. CIS is implemented using CCi-MOBILE in MALTAB as follows (Fig. 3)-

- The input to the computing platform running MATLAB/JAVA is the 8 ms long, 128-sample frame from CCi-MOBILE.
- The 128-sample frame is passed into an Overlap-add Block-shift window onto which a 128-point Hann window is applied, which is given as:
(1) $$w(m)=0.5\left[1.0-\text{cos}\left(\frac{2m\pi }{L-1}\right)\right]\;m=0,\ldots ,L-1$$
  The frames are shifted as per the stimulation rate chosen by the researcher (default- 1000 pulses per second (pps)).
- Next a 128-point Fast Fourier Transform (FFT) is performed, which yields bin frequencies (f_c_) linearly spaced at multiples of 125 Hz. As the input signal is received in real-time, the output is Hermitian symmetric and only bins 1 to 64 with positive frequency values are used to calculate magnitude squared spectrum as:
(2) $$X{\left(k\right)}^{2}=X_{R}^{2}(k)+X_{I}^{2}(k)\;k=1,\ldots ,L/2$$
- The squared spectrum magnitude is then scaled by a weight matrix to determine the frequency boundaries of each filter channel. Filter envelope for the nth channel is given as:
(3) $$Y(n)=\sqrt{\underset{k}{\sum }a(n,k)X^{2}(k)}\;n=1,\ldots ,N$$
- Additional shape adjustment to the frequency response of the filter channel is given by a vector of variable filter channel gains *G(n)*. This makes the final processed filter bank output vector as: *Z* (*n*) = *G*(*n*)*Y* (*n*) *n* = 1*, …, N*
- The output is then scaled according to the electric dynamic range required by the CI user’s MAP parameters (maximum comfort loudness, dynamic range, and threshold values) and added to an output buffer.
- To ensure bilateral synchronization, the output buffer waits till 8 ms worth of pulses have been processed, then the buffer is passed back to CCi-MOBILE for transferring the electrode stimulation information to the implant, encoded in the biphasic pulse train.

Researchers can choose the number of electrodes active for stimulation according to their experiment requirements. If all 22 channels are kept active for the CIS strategy, then maximum stimulation rate for the biphasic pulse train can be set only up to 650 Hz (14400/22), preferably below 600 Hz (keeping the transfer rate enough below the threshold).

#### 2) Advanced Combinational Encoder (ACE) Coding Strategy:

ACE strategy was first discussed in [30] and works as an advanced adaptation of the CIS strategy, as it lessens channel interaction and prevents the risk of unnecessary or sudden activation of all electrodes simultaneously. ACE is based on the “NofM” principal of coding strategies. It stimulates fewer but most spectrally dense channels (N) per cycle than active electrodes(NofM; N<M). Previous subject tests report that in an experiment comparing the performance of ACE against CIS, subjects preferred the ACE strategy 30% more than the CIS strategy [31]. For implementation with CCi-MOBILE, ACE follows the same steps (i) to (v), after which additional steps are added before step (vi) for selecting the channels with the most spectral densities. These additional steps involve sorting the output and identifying 8–12 (“n”) out of 22 (“m”) channels that encompass the highest spectral energy in each stimulation cycle. After selecting the *N*_*maxima*_, it is logarithmically compressed using the function-

log(1+bx)/log(1+b) x=input; b=415.995

**Fig. 4.**
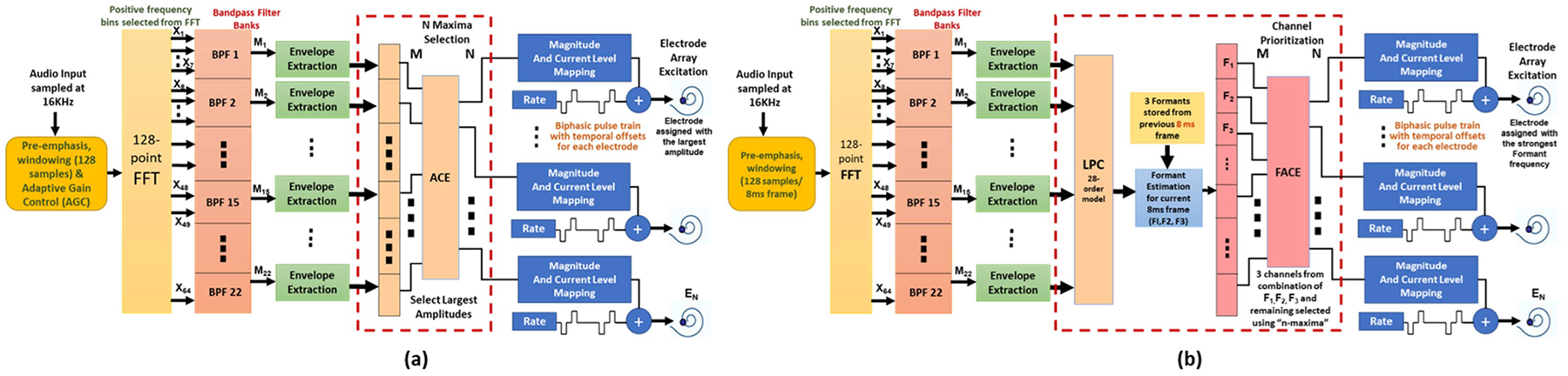
Block diagram showing (a) ACE and (b) FACE [31] implementations on the CCi-MOBILE used in the experiment.

The constant “*b*” is calculated from clinical and sound processor parameters. The compressed maxima are then scaled following the steps (vi) and (vii) of the CIS implementation on CCi-MOBILE. Fig. 4 (a) highlights the added steps for ACE.

#### 3) Formant-Based ACE (FACE) Coding Strategy:

FACE coding strategy presented in [32] which suggests additional encoding of formant frequency locations to better select the “n” out of “m” frequency channels in the ACE processing strategy. Rather than just choosing the highest spectral bands for incoming audio, choosing the spectral bands with most of the phonetic information (present in highest formant frequencies) is more beneficial as it provides both harmonic and periodic structure of speech, making it more information dense. For FACE implementation on the CCi-MOBILE, additional processing is included in the ACE strategy framework as shown in Fig. 4(b). After the step of envelope detection (common for both CIS and ACE), the channel information is passed through a 28-order linear predictive coding (LPC) block which models the overall frequency response of the speech segment for formant estimation. Three formant candidates, *F1, F2* and *F3* are estimated through this process. The current detected formant bandwidths and frequencies are compared with three formants from the previous stimulation cycle as a continuity check to ensure fluid vowel movement characterization with a 150–200 Hz bandwidth. The best 3 channels representing the combination of *F1*, *F2* and *F3* are selected and compressed at first, and the remaining channels are selected using the “*n-maxima”* criteria of ACE strategy. The chosen channels are then scaled and mapped similar to the CIS implementation.

### B. Algorithm Evaluation Protocol

Although the left and right input channels are processed independently, it is mandatory that for proper localization and speech perception, the output of both channels are effectively synchronized for BiCIs, even if different algorithms are being processed on each channel. This is essential for users who have better hearing capabilities on one ear and need extra processing on the other. For e.g., if the audiologist assigns the right ear of the BiCI user as the better ear, and the left ear to be substantially poor performing, then the left ear will need further assistance. This can be aided by having the FACE algorithm run on the left ear and CIS/ACE run on the better/right ear. In that case, a forced delay needs to be added on the processing for the right ear, to match the longer processing time of the left ear. CCi-MOBILE is designed such that it allows dynamic delay monitoring and stalls the output buffer until it has received equal amount of frame data from both left and right channel. Such a testing platform will allow audiologists to ensure better fittings for BiCIs, as they would have more information on the required delay to assist better speech understanding. A similar idea has been discussed in [33], but has not been tested with subjects.

Verifying the capability of CCi-MOBILE to process algorithms with different processing time simultaneously, and provide a synchronized output, would allow other researchers in the field to use the platform for similar experiments expanding the capabilities of CIs. To verify the performance, the two possible cases that are being considered are:

- Running the same coding strategy simultaneously on both left and right channel,
- Running different algorithms on each channel from the above-mentioned coding strategies and analyzing the processing time.

**Fig. 6.**
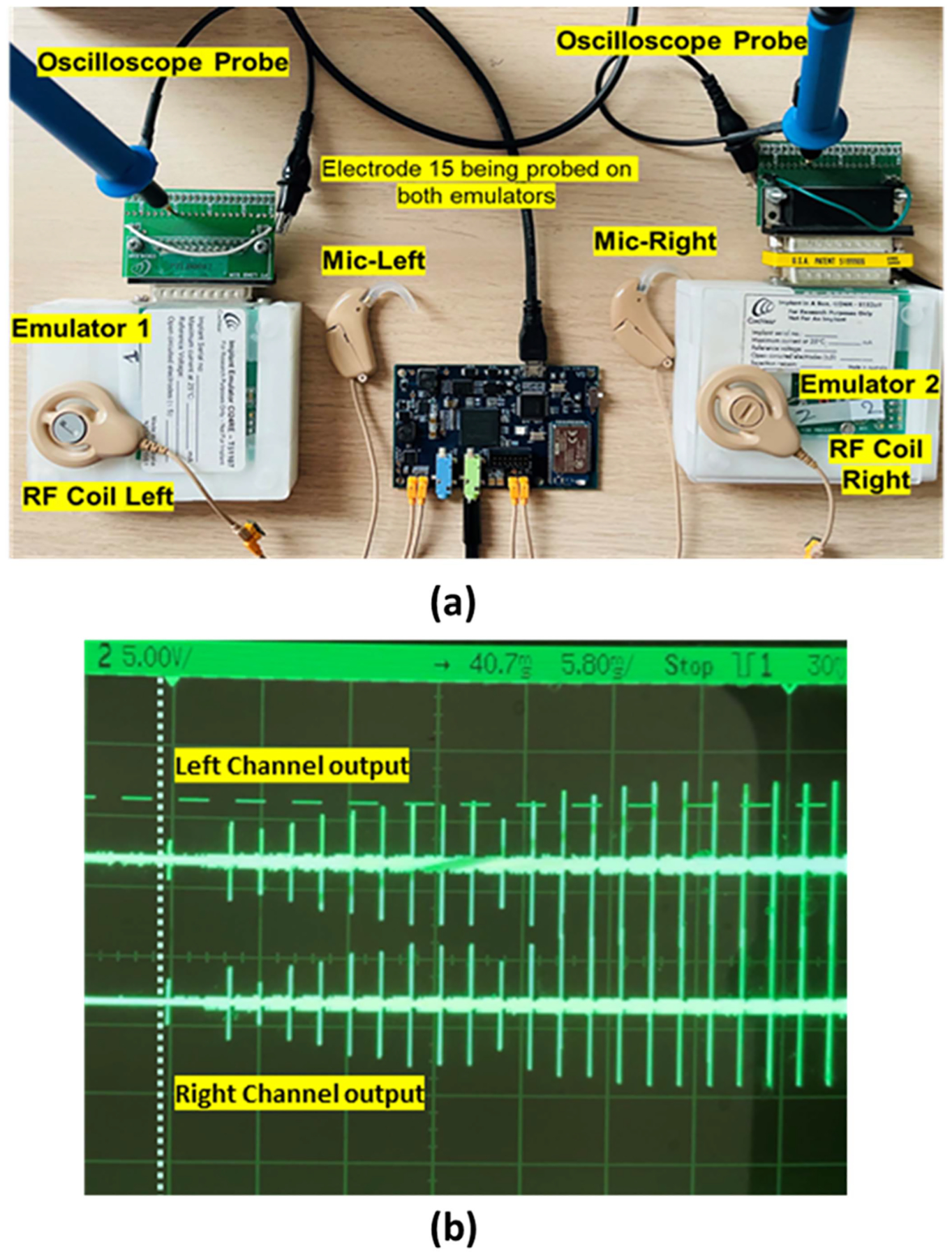
(a) The RF output of the CCi-MOBILE probed with an implant emulator and oscilloscope (b) synchronous output of the left and Right biphasic pulse train when probed at electrode 17, with an input tone of 414 Hz.

Below (Fig. 6) is the test setup for analyzing the processing time and oscilloscope output synchronization for bilateral CIs. Both ACE and CIS strategies are real-time processing strategies, whereas FACE is an offline processing strategy, as the computation time required is more than the real-time processing frame window. CCi-MOBILE is capable of effective left and right channel synchronization of both real-time as well as offline based algorithms. The processing time analysis of different coding strategies running in parallel will be discussed in the results section.

## IV. Experimental Protocol

Three experiments have been performed to compare and evaluate multiple aspects of bilateral processing by the CCi-MOBILE which are: i) simulation-based testing ii) objective testing and iii) subjective testing. The main goal of all experiments is to justify that the performance of CCi-MOBILE is not significantly different from commercially available bilateral clinical processors, and aids as the perfect substitute for independent researcher groups to test their new algorithms with subjects. Two parameters will be monitored through these experiments: i) internal throughput delay which effects the real-time processing output and ii) external delays occurring due to different directions of arrival (DoA) to each channel, assisting for sound localization.

### A. Simulation-Based Tests

#### 1) Setup:

**Fig. 7.**
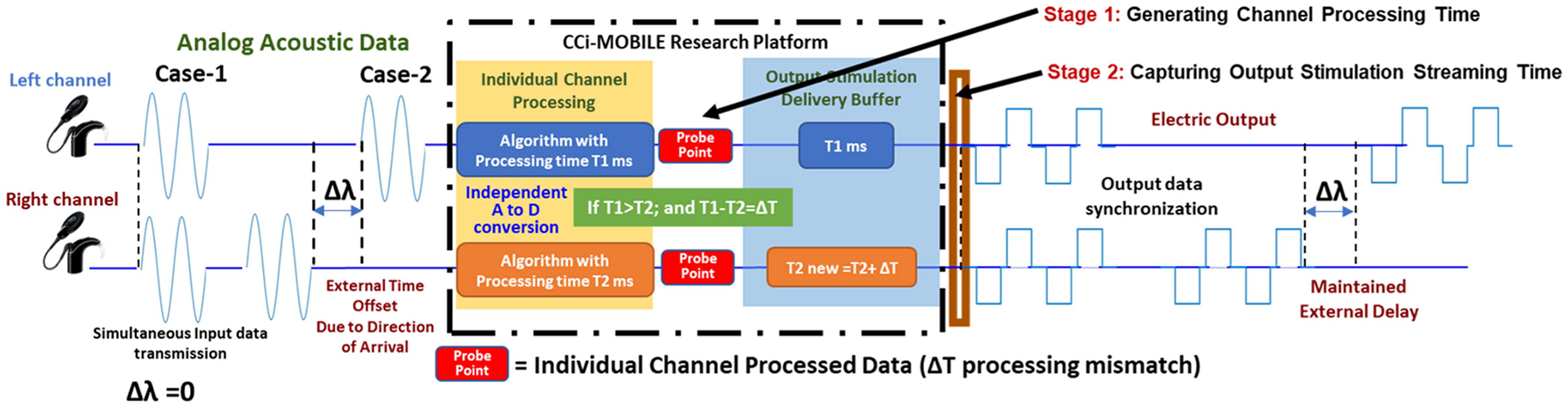
Experimental protocol for bilateral testing.

For the test set up we have left and right channels coming in; one channel may have some offset due to the difference direction of arrival and whatever processing is done on the left and right channels, we must preserve that input offset to ensure that the CI listener perceives the sound in the same direction of arrival. That would mean there should be no additional internal delay to distort the external offset. To test this hypothesis, the processing time of both the left and right channels are analyzed through two cases. The first case of experiments involves (Fig. 7, Case 1) sound coming from top dead center, hitting the mics straight, with no external delay and the processing time from both channels is probed at Stage 1 (Fig. 7). As the left and right channels utilize independent resources on the computing platform, it is possible that both channels finish processing at different times, even if it is the same algorithm. For real-time processing strategies like ACE and CIS, CCi-MOBILE should be able to stream the output with negligible delay between channels in order to match the industry made clinical processors. To ensure this happens, the output stimulation streaming buffer is programmed dynamically such that it stalls the faster processed channel till the time data is received from the slower processing channel. This dynamic channel stalling logic is confirmed with probing the Output Deliver buffer at Stage 2 (Fig. 7) and analyzing if still any mismatch exists between the channels. An example presented in the figure shows the left channel taking T1 ms to process the incoming signal, while the right channel takes T2 ms, with T1 > T2, which means the right channel processing incoming signal faster than the left channel. This creates an internal delay of ΔT; which is the ΔT processing mismatch (ΔT = T1-T2). To rectify this unwanted mismatch between channels, the output buffer should stall the right channel by adding ΔT to T2 and wait until it has received equal amount of information from the left channel. So, although at stage 1, the channels might be processing differently, at stage 2, the output streaming is monitored to ensure that the mismatch is eliminated. Being able to process algorithms independently on each channel and yet see the combined output effect of bilateral hearing is important for researchers trying to understand how to optimize fittings for users. Case 2 from Fig. 7 inserts a Δ*λ* offset that looks at the direction of arrival, which is fed into the CCi-MOBILE processing box and then we look at the left and right output streaming at Stage 2 to measure what the delta offset is obtained. A range of forced known internal offsets (between 0.05 ms to 5 ms) are randomly added to a data set of 1000 wav files (consisting of sentences, words, phonemes, and tones) to confirm whether the known offset is maintained while the output buffer is streamed through CCi-MOBILE.

#### 2) Speech Material:

Among the 1000 wav files used as a dataset for the simulation test, 250 wav files are taken from the AzBio clean database, with 125 male speakers and 125 female speakers [34]. 250 wav files mixed with 5dB speech shaped noise (SSN) from the IEEE sentences database have also been added to the dataset, again comprising of 125 male speakers and 125 female speakers. Five lists of the Consonant Nucleus Consonant (CNC) database have been added to the speech material, comprising of monosyllabic words with equal phonemic distribution across each list, with each list containing 50 words. The remaining 250 .wav files are tonal signals ranging from 100 Hz to 5 KHz each being 3 second long. Each .wav has been processed in 3 iterations, giving a total of 3000 data points to ensure accuracy.

#### 3) Experiment:

The left and right channel synchronization is analyzed through four different categories in the experimentfirst with the same algorithm on both channels; second category compares same algorithm on both channels but the stimulation is varied across the channels; third involves comparing the processing time of two different real-time coding strategies running simultaneously on each channel and calculating the throughput delay that needs to be introduced; and fourth involves analyzing the off-line algorithm performance against a real-time algorithm.

### B. Objective Parameter Analysis

**Fig. 8.**
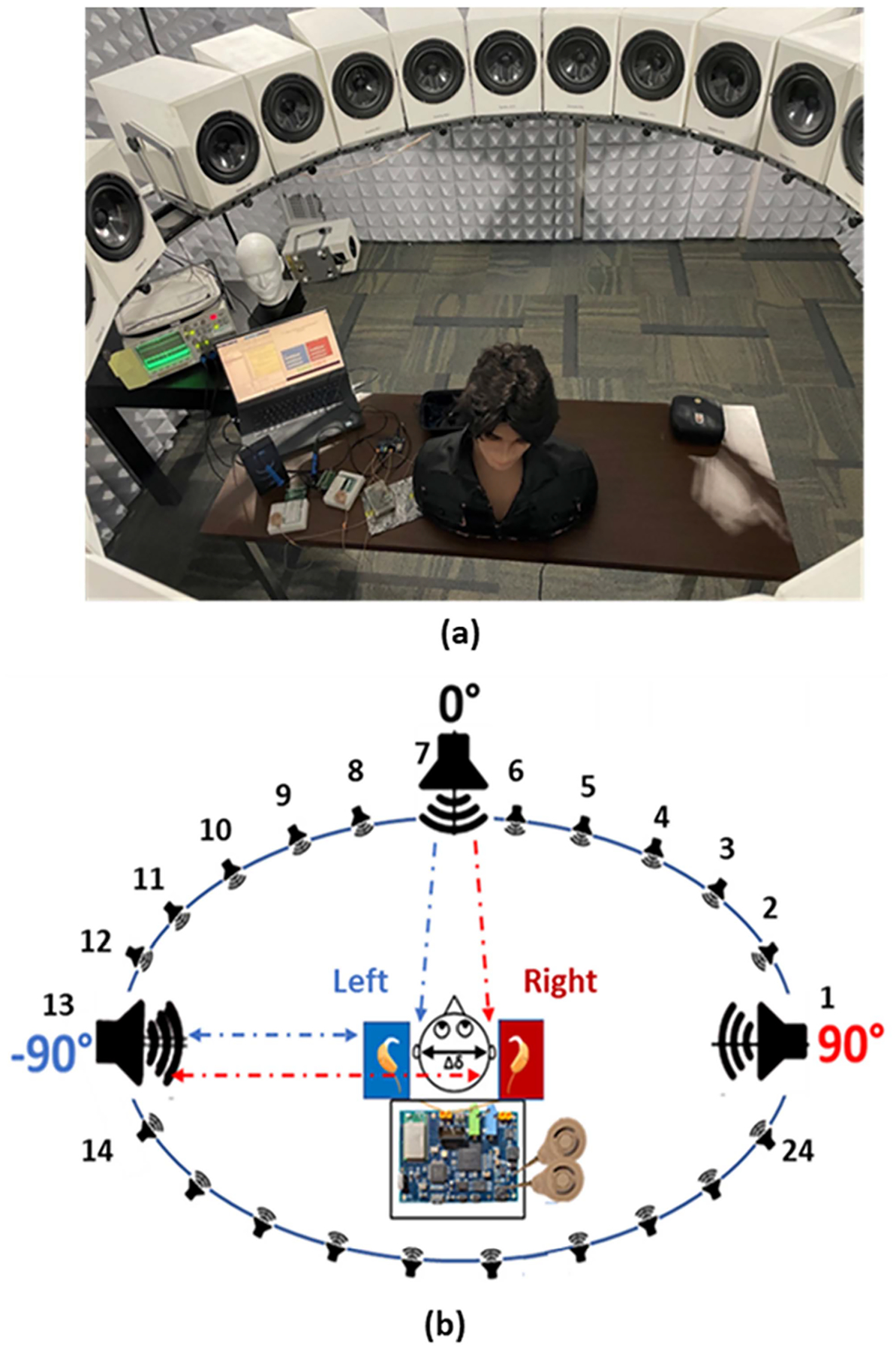
(a) 3D audio lab at callier center UT-dallas (b) objective test set-up to analyze CCi-MOBILE’s capability to localize sound real-time.

Before performing subject evaluations with CI users to confirm the hypothesis of CCi-MOBILE performing equally as commercial processors, it is important, that all aspects of system are analyzed through objectified modes of testing. This set of experiments provides validations for the case 2 described in Fig. 7(case 2) to verify the efficacy of external offsets introduced due to difference in direction of arrival. This experiment involves observing whether CCi-MOBILE is capable of capturing source localization based ITD information when real-time external delay is created in a soundproof 3D-Audio booth using a circular array of 24-loud speakers. The diameter of the 24-speaker array is 5 feet, with every speaker spaced at a 15-degree angle from each other and each consecutively numbered from 1 to 24. The loudspeakers (flat frequency response between 150 to 18000 Hz) were custom designed according to weight and size, to fit the circular mount holding the arrangement mid-air. Fig. 8 presents the arrangement of the speaker array and the overview of the experiment being performed. With speech being played at the same time from different speakers in the array, the aim is to capture the resulting delay, caused due to the difference in DoA on the oscilloscope through the RF coil output channels. The experiment protocol involved playing sounds from 3 directions-the 0° speaker, *−*90° speaker, and the +90° speaker, with the time of stimuli generation from the RF coils being captured on the oscilloscope and processing time recorded on computing platform console. As the left and right BTE mics were mounted on the ear folds of the dummy head, that replicated the exact angle in which sound would reach the bilateral CI implants, the left ear gets to be closer to the speaker at −90°, while the right ear is closer to the speaker at +90° as shown− in Fig. 8(b).So according to the hypothesis if speech is being played from the speaker *−*90 then the Oscilloscope output of the RF coil should be able to capture the left channel slightly leading the right channel by the value of ITD. Eleven cases iterated 3 times, were considered to analyze the results of audio being played from the 3 speakers in random, with clean speech as well as cases in which speech was contaminated by babble noise (BAB), speech shaped noise (SSN) and by competing speakers.

### C. Subjective Testing

In this investigation, one bilateral CI user (age: 71 years) with post lingual deafness was recruited. The subject had received a sequential implantation of the CI512 implant type from Cochlear Corporation 9 years ago. For the experiment, the subject was fitted bilaterally with CCi-MOBILE, and the transmission fittings were adjusted to the same MAP parameters received from the audiologist. The subject has been named ‘CI-01’ for this experiment. The loudness levels were set to the subject’s comfort through the CCi-MOBILE MATLAB GUI. Testing was performed only in bilateral mode with the CCi-MOBILE. Unilateral processing performance has been previously discussed in [25]. The subject was tested in the same 11 case conditions that we mentioned for objective testing. The main task was to identify the speaker number of the source in presence of interferers, such as noise or competing speakers. For e.g., if speaker 1 (at +90°) played the source audio, speaker 7 (at 0°) would play a SSN noise, and the subject had to identify the source signal and the noise. The main objective of this experiment was to analyze through subject tests whether CCi-MOBILE is capable to capture and deliver small precisions of sound localization cues, allowing researchers the capabilities to test more advanced bilateral algorithms involving ITD and ILD information.

## V. Simulation Based Results

The bilateral channel simulation-based results as mentioned before, have been analyzed in 3 different categories. The processing time as well as the output buffer streaming times have been compared for various sub-cases. The results are for cases where both the left and right channel are active.

### A. Realtime Processing of Algorithms on Both Channels

**Fig. 9.**
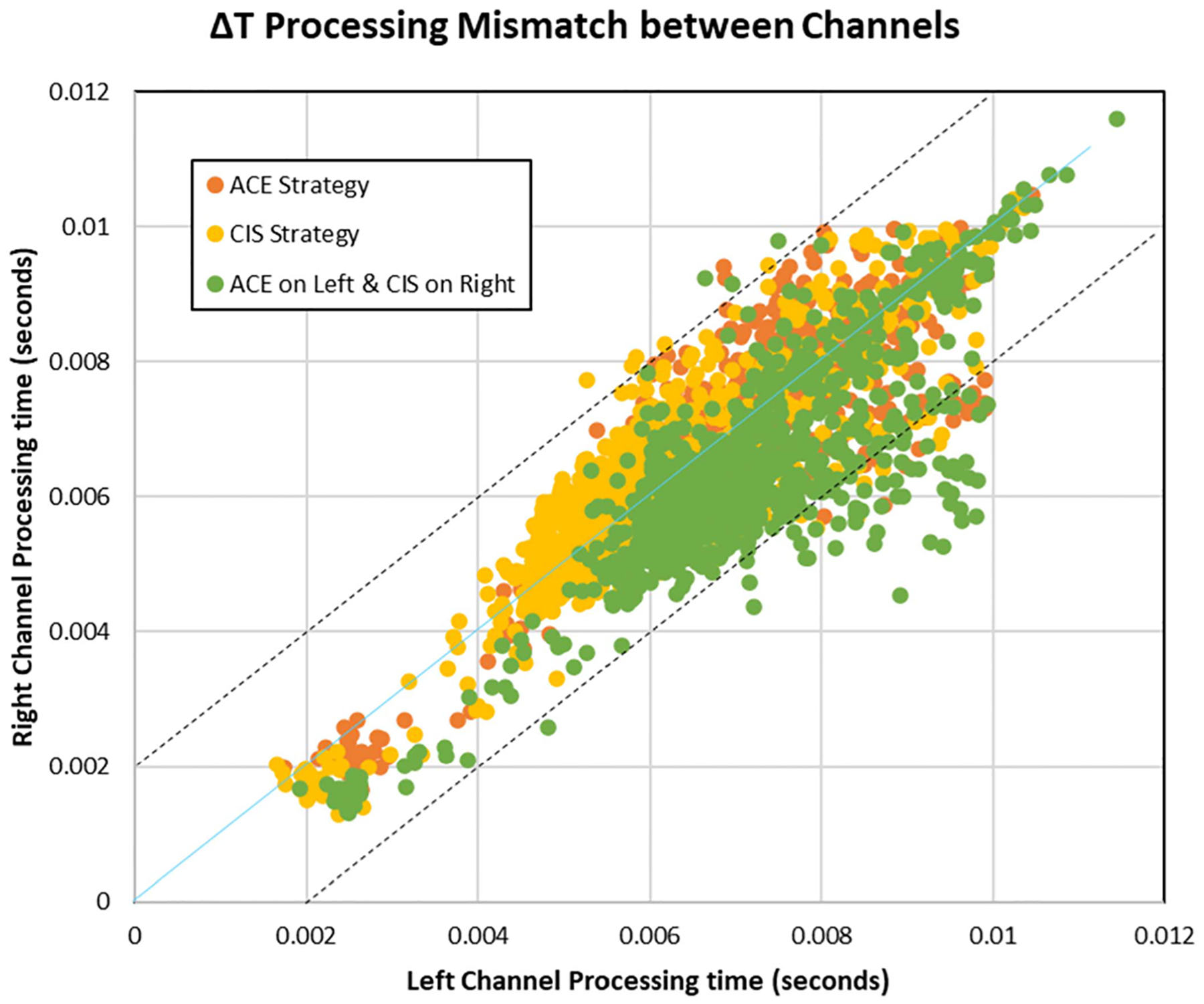
Comparing processing time of ACE, CIS, and ACE+CIS on left and right channel simultaneously at stimulation rate of 500 pps.

Fig. 9 shows the x-y scatter plot for the left and right channel processing times of real-time incoming audio. The left channel is plotted along the x-axis while the right channel processing time is plotted on the y-axis and 3 cases of real-time processing are color coded. The orange x-y scatter plot presents the processing time results when both the left and right channel generate stimulations through ACE coding strategy. It can be noticed that as the progression of the values are along the diagonal, for a given value of x (left channel), y (right channel) has almost the same values (apart from a few outliers), which means that the parallel processing happens simultaneously on both channels for the most part. The average processing time taken by ACE strategy is 6.93 ms which equals to the throughput. Processing time for CIS strategy is color-coded in yellow, and as CIS performs very similar to ACE strategy, in Fig. 9 most of the value points have got overlapped. The processing times for the left and right channel using the CIS strategy followed the same trend in the x-y scatter plot as Ace, with both channels processing the incoming real-time data at the same time. The average processing time through the CIS strategy is 6.08 ms. The third legend demonstrated in Fig. 9, color-coded in green is a mixed strategy implementation, where ACE is running on the Left channel and CIS on the right. As it can be seen that this mixed processing does not have much of the linear trend and most of the values do not fall on the diagonal, rather they fall below the diagonal. This means that most of the time, CIS on the right channel processed the data faster than the Left channel with ACE, which is also justified by the fact that the average processing time with CIS is lesser than that through ACE strategy. But as the processing difference between the two channels still remains lesser than 5 ms throughout, the output buffers are able to stream the output in real-time. Also, in this section, all the strategies were evaluated with a stimulation rate of 500 pps. It is important to know that after processing data independently, each channel transfers the processed information to the left and right output buffers respectively. The buffer that gets filled faster waits for the other buffer to receive equal amount of information and then the output from both buffers is streamed synchronized with the output clock. The output data buffer streaming time will be discussed in further section to validate the output synchronization.

### B. Processing Time Analysis With Varying Stimulation Rates

**Fig. 10.**
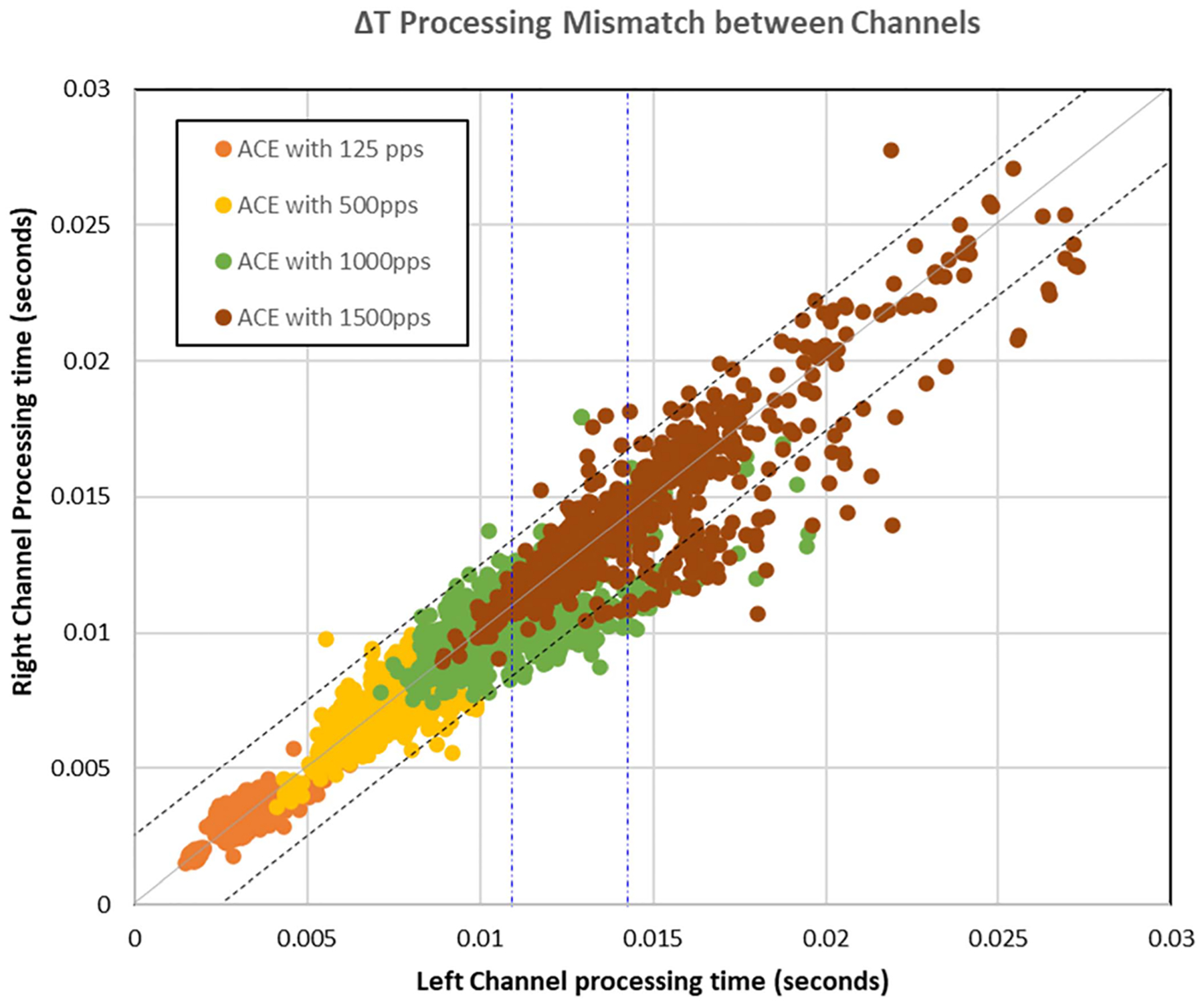
Comparing processing time of different stimulation rates.

The second out of the 3 categories being investigated for channel processing time was analyzing how the rate of pulse stimulation affects the processing throughput. For this experiment, processing through ACE strategy was kept constant on both channels. Four different stimulation rates- 125 pps, 500 pps, 1000 pps and 1500 pps were analyzed. Fig. 10 presents the processing throughput when the same 1000 .wav files were given as input, with all the stimulation rates mentioned above. The figure confirms the hypothesis that lesser stimulation rate, lesser is the processing throughput. The legend color coded in orange shows the processing time taken when the stimulation rate is maintained at 125 pps, which is the minimum possible stimulation rate using CCi-MOBILE for safety reasons. The average throughput on both channels is 3.15 ms, with almost simultaneous processing. The yellow coded data points represent data being processed through ACE at the stimulation rate of 500 pps and as discussed in the previous section the average throughput of processing data at 500 pps resulted in a higher processing delay 6.93 ms, which is almost double the processing time with the 125 pps stimulation rate. A similar trend was seen when the stimulation rate of 1000 pps was used for processing the 1000 .wav files, which is presented as the green data points in Fig. 10 which had an average throughput of 10.05 ms. When the stimulation rate, was kept at a high of 1500 pps, quite a few outliers were observed, with mismatch in processing time of the individual channels, with a higher average throughout of 13.96 ms. Although there are numerous outliers for the 1500 pps stimulation rate case, the output buffers will be stalling the less filled buffer to support the synchronized normal hearing like experience.

### C. Input Versus Output Buffer Streaming

It is known that as bilateral CI users have separate implant systems on both ears with no form of connection between the two processors, it becomes next to impossible to provide the best suited fittings such that the combined sound experience becomes more human like. CCi-MOBILE allows the freedom of independent processing on individual channels but also allows researchers to fine tune the output by stalling the faster processing channel in the output buffer, such that the RF coils stimulate biphasic pulses in a synchronized form when there is no external delay. One of the major challenges that CCi-MOBILE was aimed at overcoming when compared to previous research platforms was, the output synchronization of channels against the same output clock. No matter which channel processes incoming data faster, the output buffer does not release the biphasic pulse stream until it has equal left and right data. This ensures that no internal delay can tamper the RF coil output, such that when external delays due to difference in DoA occur, the platform can effectively capture and transmit the precise external delay information.

**Fig. 11.**
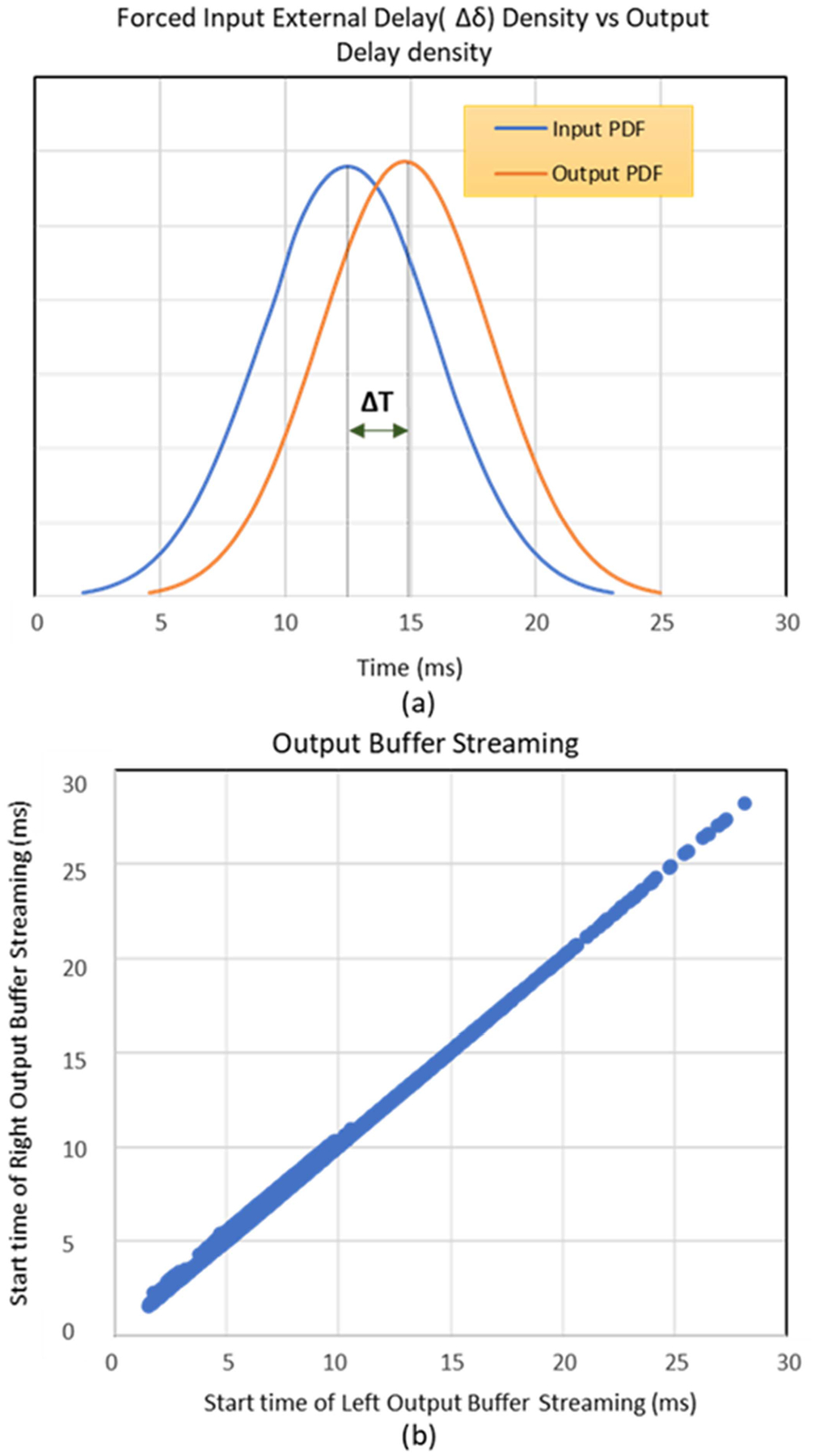
(a) Input vs output pdf with external delay added at input. (b) x-y scatter plot visualizing the relation between the left and right output buffer streaming.

In this experiment, a sanity check has been made to ensure that any for a known forced delay set at the input, the same delay should be maintained at the output as well. The input wave files were added with a known external delay between 5 ms to 20 ms, and the output buffer streaming start time was recorded. All the cases discussed before are included in this dataset: ACE on both channels; CIS on both channels; ACE on left channel +CIS on right channel; ACE at 125 pps; ACE at 1000 pps; ACE at 1500 pps. The delays chosen, were randomly added to the input and then the input time density was compared to the output density data points. Fig. 11(a) shows the bell curves for the input vs output density functions (pdf), with both formed parallel to each other. ‘ΔT’ is the throughput value for each input which on an average is 6 ms for a given input. The pdf comparison confirms the hypothesis that any kind of external delay information provided at the input is maintained at the output as well, ensuring the integrity and synchronization of both the left and right channel. With every case contributing 1000 datapoints on its own, the total datapoint points for this analysis is 6000. It can be seen from Fig. 11(b) that no matter whether one channel processes an incoming signal faster than the other, the output buffer streams, both channel data at the same time. All left and right output datapoints are synchronized, as the x-y scatter plot falls almost on the linear diagonal axis, with the streaming time starting from 2 ms after receiving the input to 28 ms for inputs with added external delay.

### D. Processing Algorithms With Higher Throughput Difference

**Fig. 12.**
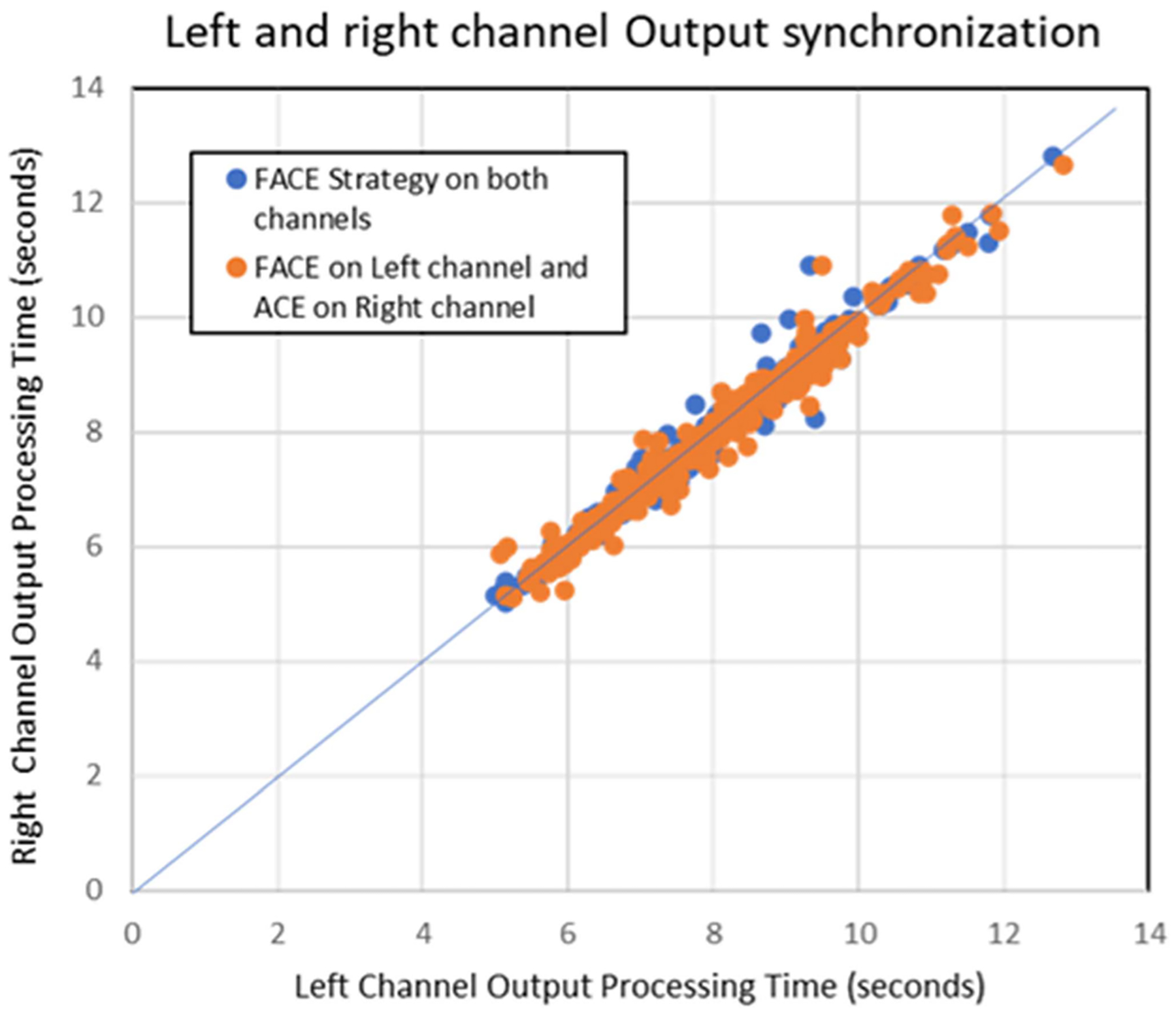
Comparing processing time of different stimulation rates.

Although it has been mentioned before that the output buffer stalls until all the left and right channel information have been filled, this experiment presents a condition for algorithm with longer throughput delay. Algorithms that take more than 1 second of throughput processing time, cannot be evaluated in real-time, but if researchers plan to test heavy processing algorithms on each channel and wish to see the combined results, CCi-MOBILE allows testing for such varying levels of algorithm performance. Fig. 12 shows the results with FACE output streaming on both channels (color coded in blue) along with the results of the signal processed by FACE on the left channel while output streamed through ACE on the right channel (orange coded data points). With FACE process having an average processing time of 7.71 seconds, the ACE output streaming (avg. processing time 6.91milliseconds) got stalled by 7.703 seconds, maintaining the integrity of the platform. This would lead to an overall off-line based algorithm processing. Such experiments involving advanced noise cancelling-algorithms or speech enhancement can be used to perform psycho-acoustic testing in off-line bilateral mode with the CCi-MOBILE for subjects with varying levels of hearing impairment in each ear.

## VI. Objective Test Results

**Fig. 13.**
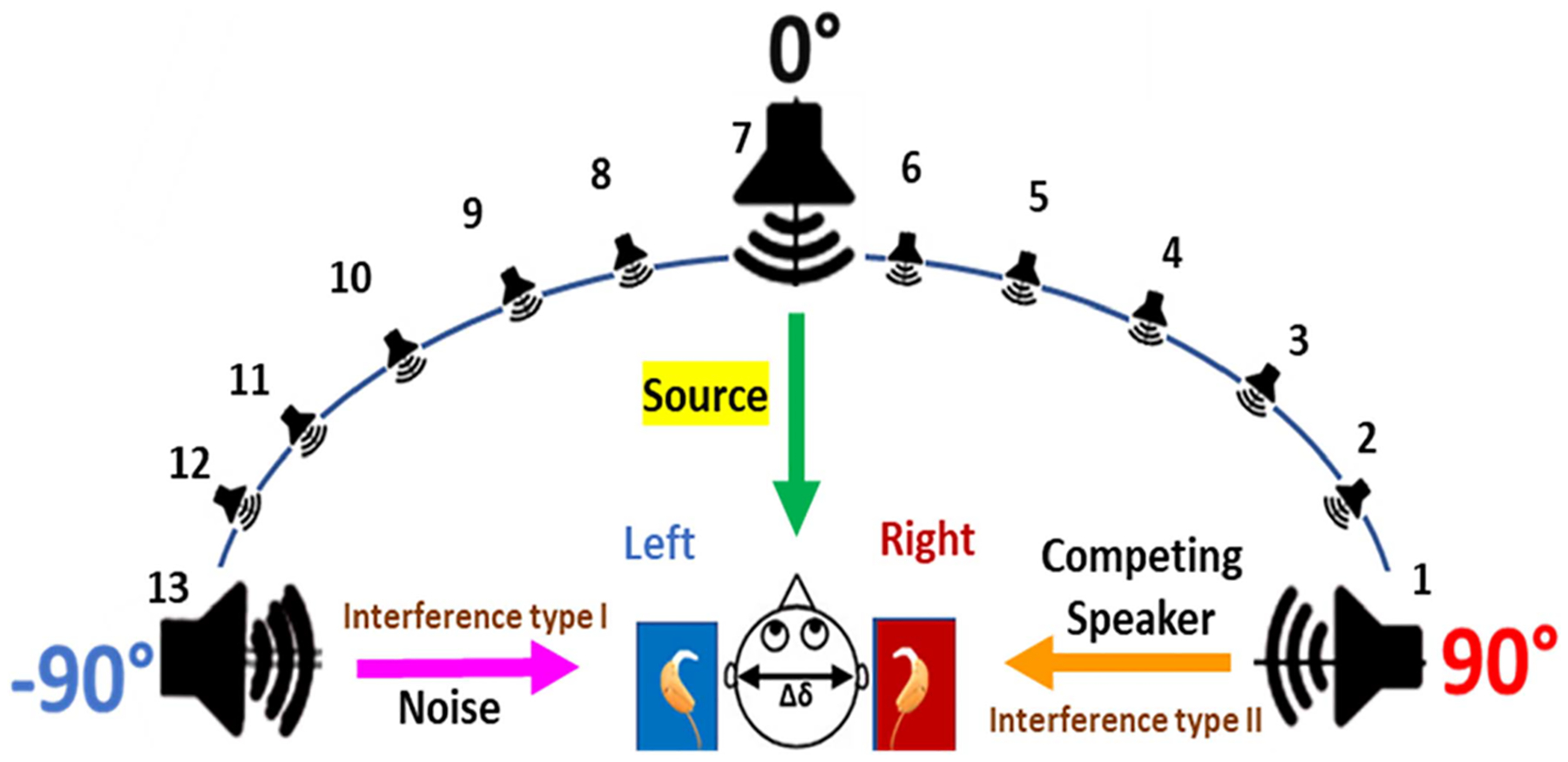
Color coding for the source, noise and competing speaker.

This section discusses the results of the final RF coil output as seen on the oscilloscope in the form of a stream of biphasic pulses. The hypothesis here is, if audio played from the circular speaker array is reaching both the left and right BTE microphones at the same time, the biphasic output should superimpose on each other, with no delay between the output channels as, that would mean, the audio sound is localized to the front or back of the listener. Fig. 13 presents the 3 active speakers for the experiment (*−*90°, 0° and +90°) along with the color coding of the source or interference being played.

**Fig. 14.**
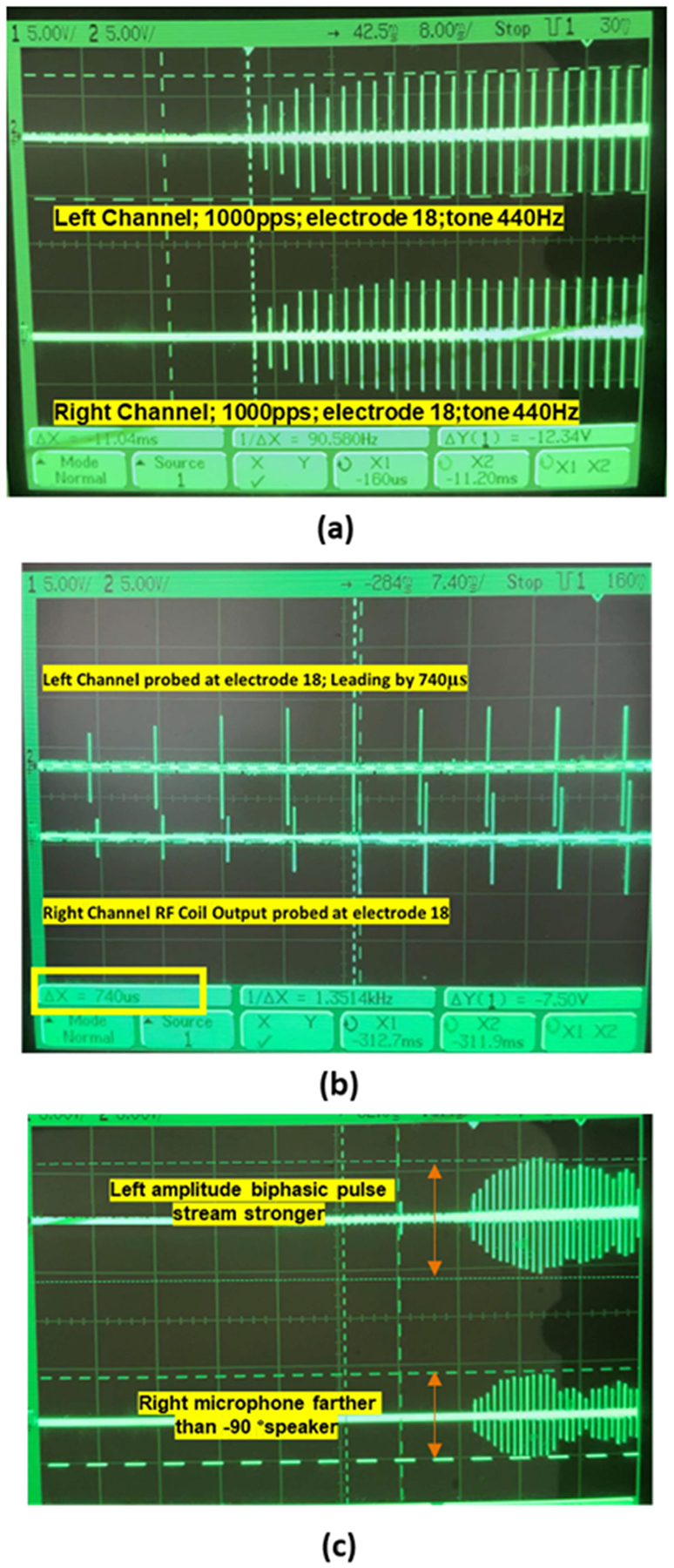
(a) Sound played form 0° speaker giving simultaneous synchronous output (b) sound played from the *−*90° speaker giving the left channel a lead of 740 μs. (c) Amplitude difference in channel, when source is closer to the left input (*−*90° speaker) and the noise is played from the 0° speaker.

The CCi-MOBILE has been tested for 11 cases of incoming sound direction. A very important factor for which the CCi-MOBILE is tested for is, its performance in the presence of interference and additional noise apart from the source. As mentioned before SSN, BAB and competing speaker type interferences were chosen for this experiment. Fig. 14(a) confirms the synchronous simultaneous output streaming when sound is coming directly from the front (0° speaker). Fig. 14(b) shows the oscilloscope results of a case where, the source sound is being played from the *−*90° speaker and BAB noise is being played from the 0° speaker. Both the left and right output channels are probed on electrode 20 and a tone signal of 440 Hz is being played as the source signal from speakers 7 (–90° speaker) and babble noise (BAB) is played from speaker 1 (0° speaker). It can be seen that there is a 740 μs lead (ΔX value from figure) on the left channel, highlighting that the left ear received the signal first, which is a cue to the brain that the sound is coming from the left direction. This scope results shows that CCi-MOBILE is capable of capturing small precision of localization information ITD. Fig. 14(c) shows that CCi-MOBILE can capture amplitude difference in channels, which also supports the fact that difference in loudness levels and amplitude can detect directional properties for bilateral CIs through ILD information.

## VII. Subjective Test Results

This section presents results of CCi-MOBILE’s performance in source localization through subjective test results with subject CI-01. Five normal hearing (NH) subjects were also recruited to quantify the performance of source localization by CI users versus NH individuals. Fig. 14 highlights the 11 cases which were considered for the experiment. The average root mean square (RMS) error values in source localization were calculated in each case for all the subjects. RMS error calculation is a standard measure for evaluating sound localization correctness across a circular array. All the subjects had to detect the source in presence of unwanted interference, which is a case that has not been explored much. The average RMS error in source detection for the CI subject was calculated to be ±9.54°, which when compared to prior research involving Bilateral CI users using their own processors is a good result [35]. This means that the subject was able to detect the direction of the source but was off by about 9° from the exact location, which is acceptable. The average RMS error performance of the NH subjects was ±2.87°, which was expected, due to the natural hearing mechanism of ITD and ILD support. The CI subject test confirmed that CCi-MOBILE is capable of delivering ITD and ILD based directional cues if researchers are interested in testing such specific algorithms.

## VIII. Conclusion

BiCIs enable more of a normal hearing like experience to its users as it provides access to time, level and spectral differences between incoming sound signals when the sound hits both CIs. But synchronizing the fittings for both ears to generate an effective combined effect needs optimized fitting and interaural matching of electrodes. Being able to add, control and test ITDs and ILDs contributes to such optimized fitting, for which the developed bilateral algorithms need to be tested by varying multiple parameters, preferably on a research platform. A set of modified bilateral algorithms and their performance on the CCi-MOBILE testing platform has been discussed in this work, that can help signal processing researchers in the CI/HA field to accelerate the advancement and optimization their own algorithms. Both the objective and subjective results present that CCi-MOBILE is capable of capturing small precisions of external delay caused due to source localization, which is the main requirement while developing bilateral algorithms while replicating the performance of clinical processors itself. The simulation results confirmed the validity of the CCi-MOBILE research platform for bilateral processing from the computing platform perspective. A bilateral coding strategy discussed in [24] also uses CCi-MOBILE as a validating platform for a newly developed bilateral CI algorithm, which showed slight improvement in source localization performance through subject tests. Although it can take some time for CI users to get accustomed to newer algorithms, with continuous work being done to replicate the natural binaural processing, CCi-MOBILE can be used for testing such future modifications and play an important role in supporting algorithm development in the CI field.

## Figures and Tables

**Fig. 5.**
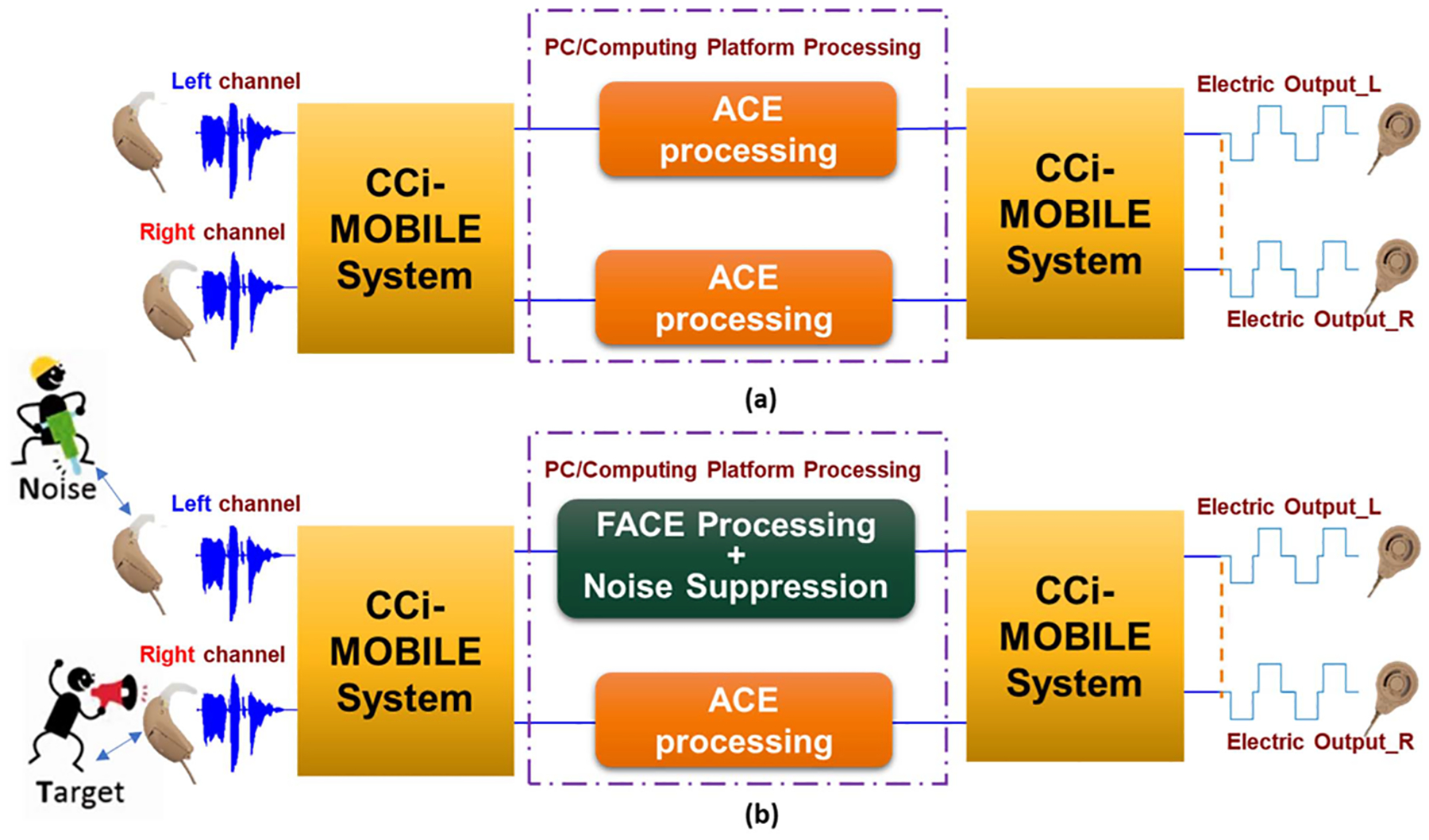
(a) Processing of same algorithms on both channels (b) processing two different algorithms on BiCI to support varied environmental conditions.

**Fig. 15.**
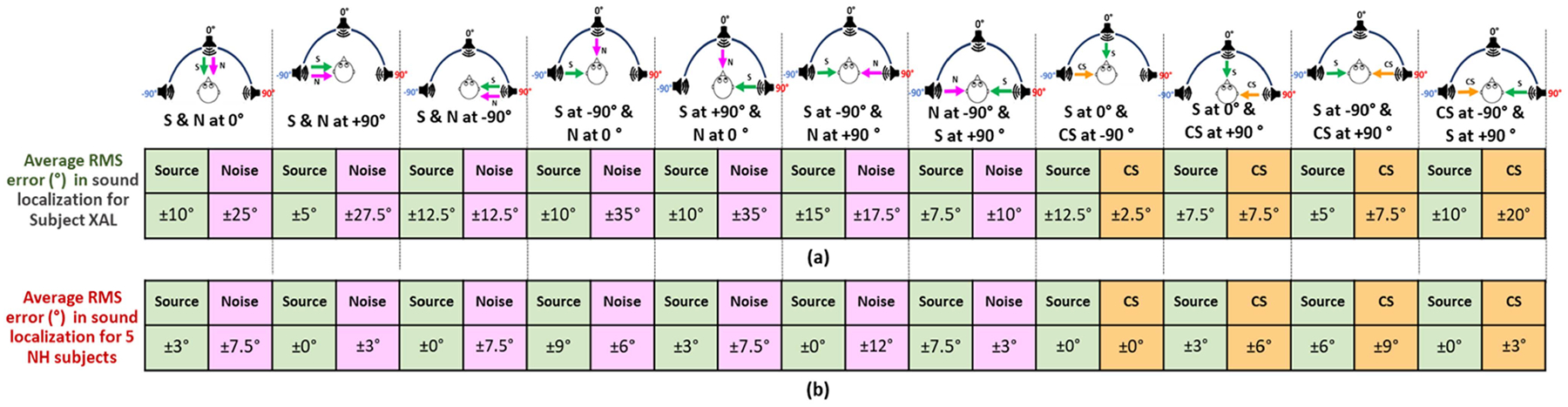
(a) Average RMS error results for CI subject CI-01 (b) average RMS error results for 5 NH subjects.

## Footnotes

1 The Nucleus series is an implant system developed by Cochlear Corporation which is the largest manufacturer of CIs worldwide.
